# Canine Population Structure: Assessment and Impact of Intra-Breed Stratification on SNP-Based Association Studies

**DOI:** 10.1371/journal.pone.0001324

**Published:** 2007-12-19

**Authors:** Pascale Quignon, Laetitia Herbin, Edouard Cadieu, Ewen F. Kirkness, Benoit Hédan, Dana S. Mosher, Francis Galibert, Catherine André, Elaine A. Ostrander, Christophe Hitte

**Affiliations:** 1 Cancer Genetics Branch, National Human Genome Research Institute, National Institutes of Health, Bethesda, Maryland, United States of America; 2 CNRS UMR6061 Génétique et Développement, Université de Rennes 1, IFR140, CS 34317, Rennes, France; 3 The J Craig Venter Institute, Rockville, Maryland, United States of America; University of Montreal, Canada

## Abstract

**Background:**

In canine genetics, the impact of population structure on whole genome association studies is typically addressed by sampling approximately equal numbers of cases and controls from dogs of a single breed, usually from the same country or geographic area. However one way to increase the power of genetic studies is to sample individuals of the same breed but from different geographic areas, with the expectation that independent meiotic events will have shortened the presumed ancestral haplotype around the mutation differently. Little is known, however, about genetic variation among dogs of the same breed collected from different geographic regions.

**Methodology/Principal Findings:**

In this report, we address the magnitude and impact of genetic diversity among common breeds sampled in the U.S. and Europe. The breeds selected, including the Rottweiler, Bernese mountain dog, flat-coated retriever, and golden retriever, share susceptibility to a class of soft tissue cancers typified by malignant histiocytosis in the Bernese mountain dog. We genotyped 722 SNPs at four unlinked loci (between 95 and 271 per locus) on canine chromosome 1 (CFA1). We showed that each population is characterized by distinct genetic diversity that can be correlated with breed history. When the breed studied has a reduced intra-breed diversity, the combination of dogs from international locations does not increase the rate of false positives and potentially increases the power of association studies. However, over-sampling cases from one geographic location is more likely to lead to false positive results in breeds with significant genetic diversity.

**Conclusions:**

These data provide new guidelines for association studies using purebred dogs that take into account population structure.

## Introduction

The domestic dog species (*Canis familiaris*) is divided into over 300 pure breeding populations known as breeds. Many breeds are characterized by reduced genetic diversity related to small numbers of founders, popular sires whose allelic pool is over represented in subsequent generations, and changes in breed popularity over time. In addition, no dog can become a registered member of a breed unless both its parents are registered members of the same breed. As a result, while phenotypic variation across breeds is large, within breed variation at the DNA level is considerably more limited than in humans [1].

In recent years, researchers have taken advantage of growing knowledge about the population structure of dog breeds [1], [2] to map genes associated with monogenetic traits (reviewed in [3]–[7]). Relying on comparative maps generated between dog and human [8], [9], the 1.5× poodle sequence [10], and, most recently, the whole genome assembly of the boxer [11], researchers have utilized families of dogs from one or a limited number of related breeds to identify loci responsible for variable phenotypes, typically associated with disease susceptibility (reviewed in [3], [4], [6]) and morphology[9], [12], [13]. As recent results demonstrate, the power to both map genes and identify causative variants is improved by comparing data from related breeds which likely share a single ancestral mutation [2], [14].

Studies suggest that linkage disequilibrium (LD) in dogs extends, on average, for megabases and varies both along the genome and between breeds [11], [15]. While this facilitates the initial mapping stages of locus identification, as only 10–30 thousand informative SNPs are needed for locus identification, it greatly expands the problem of moving from linked marker to gene, as a given LD block can extend for megabases and span over 100 genes (eg: [16]–[22]).

While some dog breeds exist in only one country or geographic region, many, such as a majority of those recognized by the American Kennel Club (AKC) [23], are distributed worldwide. Power for both meiotic linkage mapping and whole genome association studies is optimized when independent breeding populations sharing a common ancestral mutation can be considered simultaneously. Independent meiotic recombination events optimize the chance that a small region of common haplotype spanning the disease allele can be identified with strong statistical significance.

Many study designs in current use by canine geneticists address the problem of population stratification by sampling cases and controls from dogs of a single breed with little regard for the problem of population sub-structure. Worldwide sampling of pure-breed dogs sharing the same disease may increase power for both finding a locus as well as reducing LD. However it may also increase the likelihood of false positive results as population sub-structure can exist in some breeds. Indeed, the accuracy of association studies will be jeopardized if they are based on the erroneous assumption that all dogs of a single breed share the same level and type of genetic variation. Over representation of a rare allele can lead to the conclusion that a linked marker has been found when, in fact, the frequency of the allele reflects the relatedness of cases in the population. It is therefore important to determine the extent to which dog breeds may be sub-divided into smaller genetically differentiated entities, especially for cohorts sampled from different countries.

Towards that end, we have evaluated the genetic relatedness of independently bred lines of European and American dogs from the Bernese mountain dog (BMD), flat-coated retriever (FCR), golden retriever (GR) and Rottweiler (ROT) breeds. These breeds were selected based on their common susceptibility to malignant histiocytosis, a rare but highly lethal histiocytic cancer [24], [25] for which no cure or causative locus has been identified to date and for which genetic studies combining dogs from different geographic locations could be reasonably proposed. The disease is particularly common in BMD and FCR with 20% of the former ultimately succumbing to the disease [26]–[28].

Our study design utilized a set of 722 SNPs from four loci (95, 96, 260 and 271 SNPs per locus) on CFA1 each of which was genotyped in 120 dogs collected in equal proportions from the US and Europe for each of the four breeds. We analyzed the level and magnitude of diversity within each breed using allelic distribution, haplotype analysis, F_ST_ measure and clustering studies. In addition, we determined if within-breed variation was large enough to confound association studies. We studied the joint behavior of chi-square and p-value statistics for SNPs spanning a candidate region centered on a simulated causative SNP using either equal allele frequencies or allele frequencies that differ among subpopulations. Our results indicate that current sampling strategies need to take into account population substructure, particularly in breeds with high genetic diversity. When appropriately designed, the combination of samples from multiple countries or locations can offer better results and reduced false-positive rates.

## Results

### SNP genotyping analysis

Of the 120 dogs initially sampled, data was collected on 119. DNA from one US collected ROT failed to amplify for all reactions. Of the 722 SNPs selected for analysis, 43 failed to generate data in the SNPlex assay. Approximately 80% of genotypes were done in either duplicate or triplicate. The error rate, evaluated as inconstancies between duplicated genotypes, was <1%. The overall genotyping rate success was 86%. To be included in all subsequent analyses a given SNP must have had genotyping success rate greater or equal to 50% and be polymorphic, as defined by a minor allele frequency (MAF) greater than 0.05. Considering the four breeds together and the four genomic regions, 556 SNPs met these criteria.

### Breed population analysis

We calculated allelic polymorphism rate, heterozygosity rate, genetic distances, linkage disequilibrium (LD), and ultimately performed clustering methods to test the hypothesis that the four individual breeds could be separated using the polymorphism pattern of the genotyping data collected.

The GR had the greatest percentage of polymorphic SNPs (MAF>0.05) with 66.6%, whereas the BMD had the lowest with 49.0%. The ROT and FCR had intermediate values of 54.4% and 57.7%, respectively (Table 1).

**Table 1:** Allele frequencies of the 722 SNPs for the four breeds.

Breed	SNP tested	SNP with >50% of genotypes	SNP with MAF>0	SNP with MAF>0.05	Ho^5^	He^6^	% of SNP not in HWE^7^
BMD^1^	722	663 (91.8%)	409 (61.7%)	325 (49.0%)	15.1	14.8	1.5
FCR^2^	722	665 (92.1%)	455 (68.4%)	384 (57.7%)	17.1	17.9	2.4
GR^3^	722	665 (92.1%)	509 (76.5%)	443 (66.6%)	23.7	22.3	2.4
ROT^4^	722	667 (92.4%)	436 (65.4%)	363 (54.4%)	18.0	17.9	2.8

1 Bernese mountain dogs.

2 Flat-coated retrievers.

3 Golden retrievers.

4 Rottweilers.

5 observed heterozygosity.

6 expected heterozygosity.

7 percentage of SNP rejecting Hardy-Weinberg equilibrium.

The observed nucleotide heterozygosity (H_o_) rate varied between breeds, with the highest rate observed for the GR (23.7%), and the lowest for the BMD (15.1%). The H_o_ rates were close to the H_e_ (expected heterozygosity) (p<0.01), indicating no deviation from Hardy-Weinberg equilibrium for any breed (Table 1). Pair-wise genetic distances (F_ST_) between breeds were also calculated, with a mean value of 0.33. Among the six pairs analyzed, the smallest distance was observed between the two retriever breeds FCR and GR (F_ST_ = 0.237) while the BMD showed the greatest distance from any of the other breeds (F_ST_>0.34) (Table 2).

**Table 2:** Genetic distances (F_ST_) inter and intra-breed.

Breed	F_ST_ 1 inter-breed			F_ST_ 1 intra-breed (between European and US subpopulations
	BMD	FCR	GR	
BMD	-	-	-	0.048 (0.034–0.063)
FCR	0.398 (0.359–0.429)	-	-	0.03 (−0.002–0.008)
GR	0.343 (0.309–0.376)	0.237 (0.211–0.260)	-	0.07 (0.060–0.082)
ROT	0.387 (0.357–0.421)	0.346 (0.310–0.371)	0.271 (0.245–0.299)	0.03 (0.020–0.041)

1 Values are calculated based on 722 genotypes of dogs from Europe and US. Interval of 95% confidence of bootstrapping are in parenthesis.

The extent of LD was determined using the D′ statistic. For D′ = 0.5, LD values of 1.2, 1.8, 1.8 and 1.9 Mb were obtained, respectively, for GR, FCR, BMD and ROT. The extent of LD was large and not unexpected based on previous reports [11], [15]. In our study, the GR population demonstrated the shortest average LD, approximately 30% less than the three other breeds.

To determine the breed structure of the 119 samples we utilized the STRUCTURE [29] PLINK (http://pngu.mgh.harvard.edu/purcell/plink) and HCLUST (http://cran.r-project.org/) programs. STRUCTURE will group individuals into populations using either maximum likelihood methods, or by assignment of a preset number of groups (K). This program correctly divided 118 samples into four distinct breeds at the preset value of K = 4. Only one FCR clustered incorrectly and was assigned as a GR, but with a low confidence level of 0.53.

**Figure 1:**
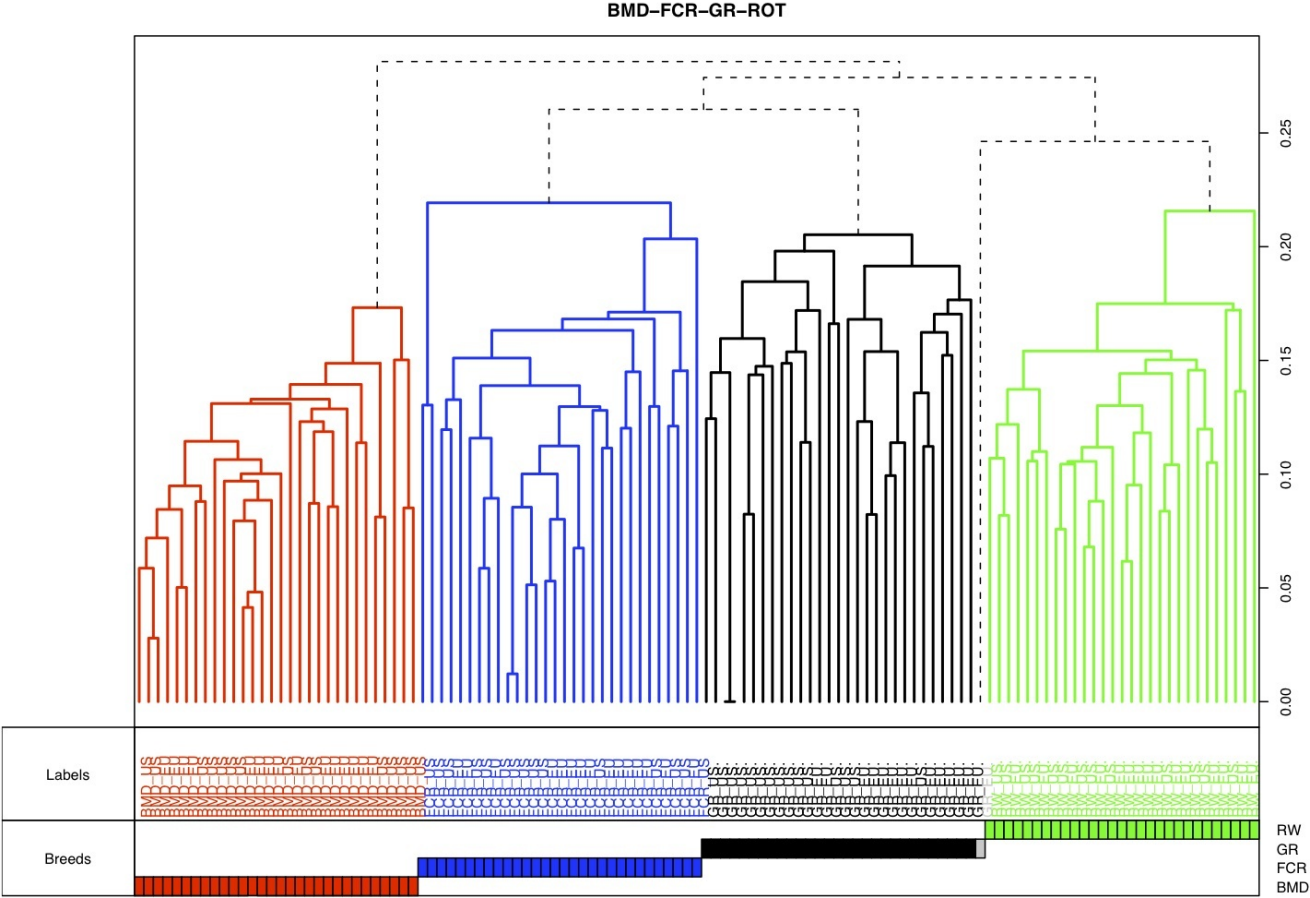
Hierarchical clustering between the four breeds. Clustering of the 119 dogs (k = 4) from the four different breeds. In the dendogram each vertical line represents a single dog and the color reflects the four clusters obtained by PLINK analysis. A grey dashed line indicates an outlier dog. Below the dendogram, dogs are named by their breed origin, and names are colored upon their cluster assignment. At the bottom of the figure, colored squares are drawn on four lines, each line representing the different breeds. The scale on the right axis represents the genetics distances calculated by PLINK software. Each breed separates from the others as the four colors correspond exactly to the four breeds, red for BMD, blue for FCR, black for GR and green for ROT.

PLINK perfectly clustered all 119 dogs, with each dog assigned to its correct breed. HCLUST divided 118 dogs into four clusters with one GR remaining as an outlier (Figure 1).

### Within breed population analysis

To explore each breed for substructure, we considered the hypothesis that a breed sampled from two geographical areas will correspond to two subsets. For each of the four breeds, the number of polymorphic SNP is very similar for both the US and European sample sets (data not shown). However, we calculated the number of SNPs for which the minor allele in one population becomes the major allele in the other population. For the GR and ROT breeds 4.1% of SNPs have allelic frequencies less than 40% in one population and more than 60% in the other population (Table S1). Only 0.9% and 0.7% of SNPs display this feature in the BMD and FCR, respectively. In addition, we examined allelic frequencies that vary more than 20% between the two populations. For the GR, 22% of SNPs fell into this category, while only 6.1%, 10% and 11% of SNPs for FCR, BMD and ROT, respectively, met this criteria (Table S2). By comparison, an average of 41.5% of SNPs demonstrated an allelic frequency difference ≥20% when considering pairs of breeds (minimum value of 39% when comparing BMD with FCR and a maximum of 43% for BMD and GR) (Table S3).

F_ST_ values were determined for each breed between the European and US populations. Although the F_ST_ values were small (mean = 0.044), the distance between US and European populations for the GR (F_ST_ = 0.07) and BMD (F_ST_ = 0.048) reflects increased genetic variation compared to the FCR and ROT, (F_ST_ = 0.03) (Table 2). As a baseline, the F_ST_ value between each breed was >0.23 (Range = 0.23–0.39).

### Haplotype analysis

To further explore the intra-breed analysis, we analyzed haplotype diversity for each locus in each population, for each of the four breeds. We first determined the haplotype blocks using the HaploBlockFinder program. The GR breed had the greatest number of haplotype blocks (n = 62, mean size = 0.61 Mb) while the BMD had the fewest (n = 36, mean size = 1.0 Mb). Within each haplotype block we then studied haplotype diversity (Table 3). The rate of shared haplotypes between US and European dogs varies only slightly for the four breeds (mean = 72.9%, range: 70.1–76.2) with the GR being the lowest (Table 3).

**Table 3:** Number of shared and specific haplotypes between the US and European subpopulations for each breed.

Breed	Total number of haploptype block	Percentage of shared haplotypes between US and EU populations	Percentage of European specific haplotypes	Percentage of US specific haplotypes
BMD	36	76.2%	11.9%	11.9%
FCR	44	73.1%	12.7%	14.2%
GR	62	70.1%	16.8%	13.1%
ROT	46	72.2%	14.2%	13.6%

### Linkage disequilibrium within breed populations

We next assessed the extent of LD for each US and European population and compared it to the complete breed data. The extent of LD between the ROTs from US and Europe were very similar (D′ = 0.5, LD of 2.1 and 2.0 Mb, respectively). When dogs from the two ROT subpopulations were combined, the results were similar with only a 5% decrease in LD (LD = 1.96 Mb). By comparison, the extent of LD for the GR between the two subpopulations is 1.4 Mb for the US subpopulation and 1.8 Mb for the European. But when GR subpopulations are combined, the extent of LD is reduced to 1.2 Mb. This 25% decrease in LD is comparable to that observed when combining two distinct breeds. This is expected as analysis of the two populations produces a shared haplotype that is, expectedly, much smaller than that observed for either population individually (Figure S1). For the BMD and FCR, when the US and European subpopulations are combined the LD decreased by only 13% and 11%, respectively.

### Within breed clustering analysis

**Figure 2:**
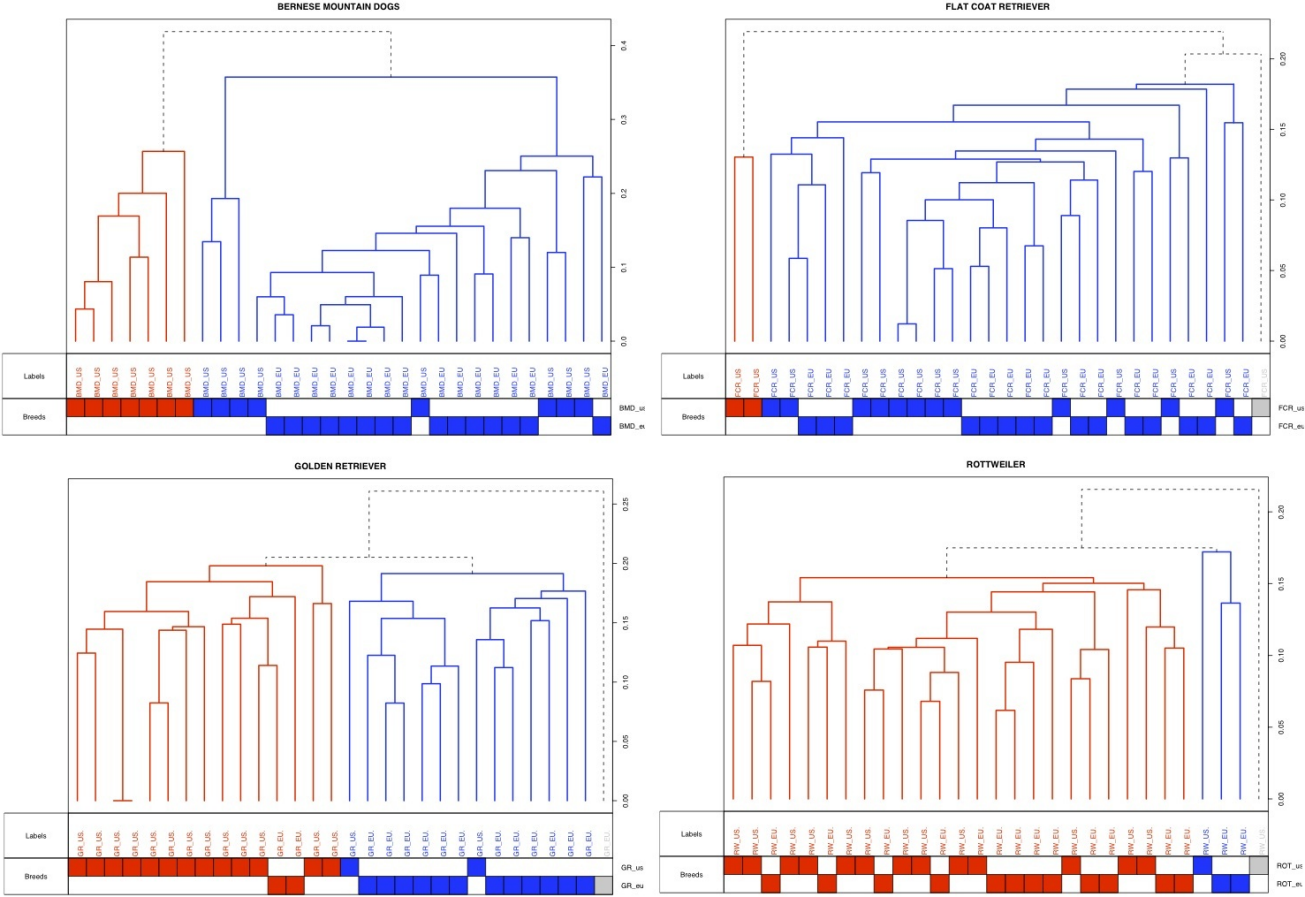
Hierarchical clustering within breeds. The figure shows the clustering analysis (k = 2) within each breed for BMD, FCR, ROT and GR (n = 30 for each breed). For each breed, a dendogram is drawn in which each vertical line represents a single dog. The two clusters are represented in blue and red, and grey dashed lines indicate outlier dogs. Below the dendogram, dogs are named by geographical origin (US or EU), and names are colored upon their cluster assignment. The scale on the right axis represents the genetics distances calculated by PLINK software.

We used the programs PLINK, HCLUST and smartpca to evaluate population substructure in each of the four breeds. No relevant clustering could be obtained for the FCR or ROT with either approach (Figure 2). However, for the GR, clustering with PLINK separated the two populations cleanly (k = 2, using SNPs with MAF≥0.10). Validity assessment of clusters is eased in our study since we possess *a priori* knowledge of the geographical origin of the dogs. In each of the two clusters (k = 2), 13 out of 15 dogs from the same geographical origin were assigned to their correct intra-breed cluster. To determine if the validity of the results depends on the clustering method used by PLINK, we used two other methods: HCLUST a hierarchical clustering method and smpartpca, a principal component analysis (PCA). FCR and ROT breeds do not show any separation of the US and the European individuals using either method. Substructure was observed for the BMD only with PCA and did not reflect exactly the country of origin: all the BMD from Europe and four US BMD have negative PC1 values, the remaining 11 US BMD have positive PC1 values (Figure S2). Substructure was also observed for the GR. With HCLUST, 13 of 15 US dogs grouped together into a cluster and 12/14 Europeans dogs separate into a second cluster with one GR remaining as an outlier (Figure 2). With PCA, 14 out of the 15 US dogs have a positive PC1 value and 14 out of the 15 European dogs have a negative PC1 value.

### Whole genome association study simulations

We have observed that a large number of SNPs have allele frequencies that vary significantly within the GR population between the US and Europe, while the same SNPs demonstrate less variation in the BMD breed. Such diversity is irrespective of disease or traits status, and within–breed population structure could influence even carefully designed studies and affect the validity of association results. To investigate the impact of within-breed genetic diversity in SNP-based whole genome association studies, we randomly selected a single SNP out of a well distributed set (n = 25 for each breed) as the causal mutation of a monogenic and recessive trait, i.e. 100% penetrance and 0% phenocopy rate. The simulated disease-causing SNPs met the following criteria: (1) dogs homozygous for the two alleles had to be found in the European and American subpopulations in order to have equal or similar proportion of the ‘causal’ genotype, i.e. four to six ‘cases’ in each subpopulation; (2) the proportion of European and American dogs for the simulated SNP had to be in Hardy-Weinberg equilibrium. In addition, the 25 chosen SNPs has to belong to different LD blocks to order avoid an artificial increase of significant p-values.

We studied the joint behavior of chi-square and p-value statistics for SNPs in the 50 Mb covered by our study. All SNPs having a MAF ≥5% were considered in the association analyses. For each breed, p-values were calculated both for the two geographical groups (n = 15 individuals each) and for the entire breed population (n = 30 individuals).

We first determined the number of SNPs with a significant p-value (<0.05) in intervals of 1, 1.5 and 2 Mb surrounding the ‘causative’ SNP. The results were similar for all four breeds. Within 1 Mb of the causative SNP, 20 to 35% of SNPs have significant p-values for the European or US subpopulation, while 32 to 44% of SNP showed significant p-values in the combined (US+European) population. We determined that p-values were one log better, from 10e-03 to 10e-04 on average, for the combined population. To assess the potential impact of allelic diversity that arises from the US and European subpopulations, we determined the number of significant p-value for SNPs located beyond 5 Mb of the causative SNP suggesting false-positives (type 1 errors) leading to spurious association. When sampled separately by continent, 5.1, 5.3, 4.1 and 3.3% of SNPs located 5 Mb beyond the simulated causative SNP had significant p-values for BMD, FCR, ROT and GR, respectively (Table 4). When the US and European datasets are combined, no inflation of false positives is observed. Indeed, we observed very similar rates of 5.9, 4.7, 3.5 and 3.7% (Table 4).

**Table 4:** Percentage of SNPs with p-value <0.05 located beyond 5 Mb of a causative SNP.

Breed	US or European population considered separately	Combined population with equal^1^ number of cases from US and EU	Combined population with unequal^2^ number of cases from US and EU
BMD	5.1%	5.9%	7.1%
FCR	5.3%	4.7%	6.2%
GR	4.1%	3.5%	7.2%
ROT	3.3%	3.7%	4.3%

1 equal or similar proportion of ‘causal’ genotype, (i.e. four to six “cases” in each sub-populations).

2 ‘causal’ genotype from one sub-population only.

Concerns regarding population stratification are particularly relevant when considering samples in which the cases and controls were not collected from the same geographic regions. We generated artificial sets of SNPs with extreme allelic differentiation bias based on geographical sampling with cases from only one continent (BMD: n = 9, FCR: n = 8, GR: n = 25 and ROT: n = 18). Causative SNPs were picked as homozygous for samples in the European or the US population in order to have 4 to 6 cases with ‘causal’ genotypes. As was done previously, we calculated p-values for all SNPs spanning the 50 Mb surrounding the simulated SNP ‘variant’. When the number of disease genotypes was unequal in the two populations, we found 7.1, 6.2, 7.2 and 4.3% for BMD, FCR, GR and ROT, respectively. The most significant change was observed for the GR, where we noted a 2-fold increase (7.2% vs 3.5%) of false positives (type I error) compared to the previous analysis, in which the number of cases and controls where equally divided in the two subpopulations. No significant increases were found for BMD, FCR and ROT.

## Discussion

Our results demonstrate that, as expected, dog breeds do not constitute homogenous entities and population stratification needs to be systematically assessed in association studies. Even within breeds with large populations, assumptions of random mating and independent allele distribution may be incorrect. Indeed, allele frequencies may vary widely both within and between breed populations irrespective of trait status with the disparities reflecting the unique genetic and social history, ancestral patterns of geographical establishment, mating practices, reproductive expansions and bottlenecks, as well as stochastic events that characterize each breed. We assessed the impact of population stratification on association studies using realistic scenarios considering purebred dogs sampled from Europe and the US. We measured the genetic variability and performed association studies with a simulated disease to assess the extent of the spurious association that can occur if unobserved population stratification exists within canine breeds or if the population structure has not been taken into account.

We used bi-allelic markers located on four unlinked genomic regions on the largest autosome (CFA1) which spans 120 Mb. Anonymous genetic markers spanning large genomic regions, such as SNPs, are considered a good indicator of the level of background diversity in cases and controls. We genotyped SNPs in four different breeds that are prone to malignant histiocystosis, including the Bernese mountain dog, flat-coated retriever, golden retriever and rottweiler.

Our results show that the GR has the highest rate of polymorphic SNPs (76.5%) and also has the greatest number of haplotypes within an LD block. These results correlate with the popularity of the breed in both Europe and the US. In the US, the GR rose in popularity in the 1930s (http://www.akc.org/breeds/golden_retriever/history.cfm) and was ranked 4^th^ among nearly 155 breeds in 2006 with more than 42,000 American Kennel Club (AKC) registrations. In France, GR was ranked 2^nd^ in popularity in 2006. Genetic diversity measures such as haplotype sharing or genetic distances (F_ST_) show that the GR has the greatest genetic variability among the four breeds studied. This breed also has the most SNPs with allelic frequency differences; 22% of SNPs have a MAF that varies by more than 20% between the two populations. Those results were supported by the fact that the GRs analyzed in this study separate significantly into two populations corresponding to their geographical origins in clustering analyses. While the US and the European GR populations have common ancestors, the popularity of this breed creates a large breeding pool on both continents and mixing between US and European populations is rare. Thus, clear population differences and associated differences in allele frequency and distribution exist in the GR that can be traced to sampling location.

The BMD has the lowest rate of polymorphic SNPs (61.7%) and the lowest number of haplotypes within an LD block. However, in contrast, they have a significant F_ST_ of 0.048 between the European and the US populations. By comparison, the F_ST_ values were negative when comparing two populations containing randomly mixed US and European samples. In addition, the PC analysis showed a substructure with all the European samples and 4 US samples in a first cluster, and a second cluster composed of the 11 remaining US samples. This result is predicted by breed history. BMD was originally developed in Switzerland in the region of Berne in 1892. Dogs were initially exported to other European countries in the 1920s and brought in the US in 1926 (http://www.akc.org/breeds/bernese_mountain_dog/history.cfm). The breed has since gained in popularity in several countries and was ranked 41^st^ in 2006 with nearly 4,000 new AKC registrations, and ranked 15^th^ in 2006 with 2900 new registrations in France (http://www.scc.asso.fr). Despite the significant increase in registrations, particularly since the 1970s, BMD are still regularly imported from Europe to the US to enhance breeding programs. The demographic history of BMD may explain why we observed a significant F_ST_ value, sub-structure was not always observed between the US and European subpopulations in the clustering analyses.

The FCR had both low allelic diversity and a low F_ST_ (F_ST_ = 0.03) and of the four breeds studied, the FCR has the lowest number of registrations in the US (551 in 2006 and ranked 100^th^by the AKC). The overall small population size of the breed severely limits the observed genetic variation within the breed, regardless of geographic origin of a particular individual.

The last breed considered in this study was the ROT, which had an intermediate allelic diversity comparable to results observed with the FCR. The ROT went through a drastic decrease of popularity in Europe at the end of the 19^th^ century, which was exacerbated during and following World War II (http://www.akc.org/breeds/rottweiler/history.cfm). In the US, the ROT is ranked 17^th^ in popularity, with 14,700 new AKC registrations in 2006. The popularity of the breed has been in steady decline since the mid 1990s, when there were more than 100,000 new registrations per year (83% decrease in the past decade) (http://www.akc.org/reg/dogreg_stats.cfm). The drastic population bottleneck that occurred after World War II and to a lesser extent the decline in popularity have limited the gene pool of ROT breed today, and may explain why its observed genetic diversity is not as high as the GR, despite the striking popularity of the ROT in the 1990s.

Our study was developed with the aim of assessing inflation of false positive results within individual breeds that could result from population stratification. By using disease simulated genotypes we have shown that type I error rate correlates with both the genetic diversity level and the sampling proportion of cases and controls between sub-populations. These results have important consequences for association studies using a population of purebred dogs sampled from multiple locations. Indeed, we did not observe a significant inflation in the false positive rate for three of the breeds in this study (BMD, FCR and ROT). Those breeds have (1) a small to medium size population and (2) a low allelic diversity between different geographical origins. In such situations, it would be advantageous to sample purebred dogs from multiple international locations to generate a larger sample size, thus increasing the overall power of the study. Indeed, we observed that p-values are one-log better, from 10e-03 to 10e-04 using a two-fold larger dataset. In contrast, the GR data demonstrated that association studies are prone to inflation of type I errors when cases are over sampled from one country. The GR have a high level of genetic diversity between sub-populations and for breeds like the GR, sampling designs need to be conservative.

Since we used a small sample size (30 dogs of each breed) to analyze simulated disease-causative SNPs, one concern is that the power and the reliability of our dataset to detect stratification may be limiting. In the GR, the sample size of 30 was sufficient to allow identification of a clear sub-structure. Although we did not detect stratification for the FCR and ROT, we cannot rule out the possibility that subtle population structure effects would have been evident had we analyzed a larger dataset or additional lineages within each breed. However if such discrete population structure exists, it would likely have little impact on association studies.

In addition to population stratification effects due to geographical origin, non random breeding based on phenotypic traits such as coat color, performance, or breed-associated behaviors such as herding, drafting and hunting may also play a role. In addition, traits such as olfaction, memory and cognitive abilities, which are partially heritable [30], may have been selected in breeds used as service dogs, leading to undetected population structure in studies involving those breeds.

### Conclusions

We have addressed the magnitude and impact of genetic diversity between four breeds sampled in the U.S and Europe and assessed the impact of within-breed population structure on SNP-based association studies. Based on this study, we recommend that researchers assess population structure through metrics such as F_ST_ values, allelic distribution, LD extent and clustering analysis. Knowing the extent of intra-breed sub-structure will increase the likelihood that results from whole genome association studies carried out on collections of dogs from single breeds are accurate. It is not possible to know all the factors that contribute to differences in allele frequency and distribution within a breed. But as these studies show, it is critical to consider such confounding effects to reduce the likelihood of erroneous conclusions.

## Materials and Methods

### DNA samples

Fifteen DNA samples from each of four breeds were collected in both Europe and the United States (120 samples total). Blood samples were collected in ACD or EDTA tubes from BMD, FCR, GR and ROT. Efforts were made to sample dogs from different breeders to minimize the chance they were themselves related to one another. For US-collected BMD, FCR, ROT and European-collected BMD dogs, pedigree data was used to verify that the dogs were unrelated to one another to at least the grandparent level. DNA from samples collected in the US was isolated using a proteinase-K, phenol-chloroform protocol [31]. DNA from samples collected in Europe was isolated using the Pharmacia-Biotech kit (BACC2), as per the manufacturer's instructions.

### SNP selection and genotyping

Samples were evaluated using single nucleotide polymorphisms (SNPs). A total of 2.5 million distinct SNPs were discovered by the canine whole genome sequencing project (http://www.broad.mit.edu/mammals/dog) by comparing reads of a boxer (7.5× whole genome assembly [11]) with a poodle (low-pass sequencing 1.5× coverage [10],) and 9 other breeds (German Shepherd, Rottweiler, Bedlington terrier, Beagle, Labrador retriever, English shepherd, Italian greyhound, Alaskan malamute and Portuguese water dog with 100,000 reads for each breed, i.e. 0.02× coverage [11]). The 722 SNPs selected from this study were mostly from within the boxer sequence or between the boxer and the 1.5× Poodle. Thus, we expect no bias in the heterozygosity statistics or other analyses. We selected SNPs from four unlinked genomic regions (termed 1, 2 3, and 4) on CFA1 at positions 7–17 Mb, 20–45 Mb, 70–85 Mb and 106–117 Mb. SNPs were selected for which both alleles were present in the boxer sequence, or alternatively, only one allele was present in the boxer sequence, with the other allele present in either the standard poodle sequence or one of the additional nine breeds. A subset of those initially selected was discarded, as they were not suitable for genotyping using the Applied Biosystems SNPlex assay system. This system was used for all subsequent genotyping as it allows simultaneous genotyping of up to 48 SNPs in one assay. Prior to SNPlex assembly sequences surrounding each variant were submitted for Blat analysis [32] to ensure that 30 base pairs flanking the SNP were unique in the canine genome. The overall SNP density was 1 SNP/50 kilobases for regions 1 and 4 and 1 SNP/100 kilobases for regions 2 and 3. Genotypes were assigned using GeneMapper software v4.0 (Applied Biosystems).

### Data analysis

Raw data statistics including the number of scored genotypes, number of polymorphic SNPs as well as number of SNPs with MAF>0.05, average observed and expected heterozygosity were calculated using Haploview software, v4.0 [33]. Population genetic distances (F_ST_) were assessed with genepop (http://genepop.curtin.edu.au/), popdist v1.1.2, using the Cavalli-Sforza and Edwards distance reconstructing measure [34] and Genetic Data Analysis (GDA) (http://www.eeb.uconn.edu/people/plewis/software.php). Clustering analyses were done using the statistical software package PLINK v 0.99q (http://pngu.mgh.harvard.edu/purcell/plink), the program STRUCTURE [35], and hierarchical clustering using the R function Hclust (http://cran.r-project.org/). Both PLINK and HCLUST use distance matrix to perform clustering. The clustering method used by PLINK will not merge clusters that contain significantly different individuals based on identity-by-state distance. For HCLUST, we used a method that merges clusters having the lowest average distances. Principal component analyses were performed using the smpartpca program included in the EIGENSOFT software [36]. Haplotypes were inferred using fastPHASE software v 1.0.1 [37] with the default settings. This software estimates the missing genotypes and reconstructs haplotypes using unphased genotype data from unrelated individuals. Haplotype blocks were determined with HaploBlockFinder program, v0.7, using SNP with MAF>0.05 [38]. Association studies simulations were conducted with Haploview_4.0beta14 and PLINK_0.99q packages.

## Supporting Information

Table S1 Percentage of SNPs with variation of allele frequencies for each breed. 1: percentage of SNP (and number in parenthesis) for which allelic frequency varies more than 10% between US and EU population. 2: difference in allelic frequency more than 20%.
(0.02 MB DOC)

Table S2 Percentage of SNPs with allele frequencies that vary more than 20% between breeds 1: Actual number of SNPs is in parenthesis
(0.02 MB DOC)

Table S3 Percentage of SNPs with switch from major allele to minor allele in each breed 1: Bernese mountain dog, 2: Flat-coated retriever, 3: Golden retriever, 4: Rottweiler, 6: Number of SNP for which there is a change from minor allele (MAF<0.5) to major allele (MAF>0.5) in percentage and in number of SNP in parenthesis, 7: change from minor allele with MAF<0.4 to major allele with MAF>0.6 and vice-versa in percentage and in number of SNP in parenthesis.
(0.03 MB DOC)

Figure S1 Using subtle population sub-structure to shape shorter-range patterns of haplotype and LD. Individuals in isolated dog populations (US or EU) with few or no exchange between them lead to a specific haplotype pattern and extent of LD. Population merged from both US and EU continent generated shorter-range patterns of haplotype, and thereby create shorter-range LD.
(0.04 MB JPG)

Figure S2 Principal component analyses were performed with the smartpca program from the Eigenstrat software. Each breed were analyzed separately and the PC1 and PC2 scores of each dog were plotted. Red squares represent European dogs and blue diamond US dogs.
(0.18 MB TIF)
