# Supplementary material for: Canine Population Structure: Assessment and Impact of Intra-Breed Stratification on SNP-Based Association Studies
Source: PLoS One. 2007 Dec 19;2(12):e1324. doi: 10.1371/journal.pone.0001324 (PMC2129117; doi:10.1371/journal.pone.0001324)
Supplement: Table S2 — Percentage of SNPs with allele frequencies that vary more than 20% between breeds 1: Actual number of SNPs is in parenthesis (0.02 MB DOC) [file pone.0001324.s002.doc]

Table S3 :

Percentage of SNPs with allele frequencies that vary more than 20 % between breeds

| Breed | BMD | FCR | GR | RW |
| --- | --- | --- | --- | --- |
| BMD |  | 39,01% (254)^1^ | 43,01% (280) | 42,63% (278) |
| FCR |  |  | 40,86% (266) | 41,87% (273) |
| GR |  |  |  | 42,02% (274) |
| RW |  |  |  |  |

1: Actual number of SNPs is in parenthesis.
