# Supplementary material for: Canine Population Structure: Assessment and Impact of Intra-Breed Stratification on SNP-Based Association Studies
Source: PLoS One. 2007 Dec 19;2(12):e1324. doi: 10.1371/journal.pone.0001324 (PMC2129117; doi:10.1371/journal.pone.0001324)
Supplement: Table S3 — Percentage of SNPs with switch from major allele to minor allele in each breed 1: Bernese mountain dog, 2: Flat-coated retriever, 3: Golden retriever, 4: Rottweiler, 6: Number of SNP for which there is a change from minor allele (MAF<0.5) to major allele (MAF>0.5) in percentage and in number of SNP in parenthesis, 7: change from minor allele with MAF<0.4 to major allele with MAF>0.6 and vice-versa in percentage and in number of SNP in parenthesis. (0.03 MB DOC) [file pone.0001324.s003.doc]

Supplementary table 1: Percentage of SNPs with switch from major allele to minor allele in each breed

| Breed | switch 50-50^5^ | switch 40 to 60 and 60 to 40^6^ |
| --- | --- | --- |
| BMD^1^ | 4,61% (30) | 0,9% (6) |
| FCR^2^ | 5,07% (33) | 0,7% (5) |
| GR^3^ | 12,3% (82) | 4,06% (27) |
| RW^4^ | 8,90% (58) | 4,14% (27) |

1: Bernese mountain dog, 2: flat-coated retriever, 3: golden retriever, 4: rottweiler, 6: Number of SNP for which there is a change from minor allele (MAF<0.5) to major allele (MAF>0.5) in percentage and in number of SNP in parenthesis, 7: change from minor allele with MAF < 0.4 to major allele with MAF > 0.6 and vice-versa in percentage and in number of SNP in parenthesis
