# Supplementary figures and images for: Canine Population Structure: Assessment and Impact of Intra-Breed Stratification on SNP-Based Association Studies
Source: PLoS One. 2007 Dec 19;2(12):e1324. doi: 10.1371/journal.pone.0001324 (PMC2129117; doi:10.1371/journal.pone.0001324)

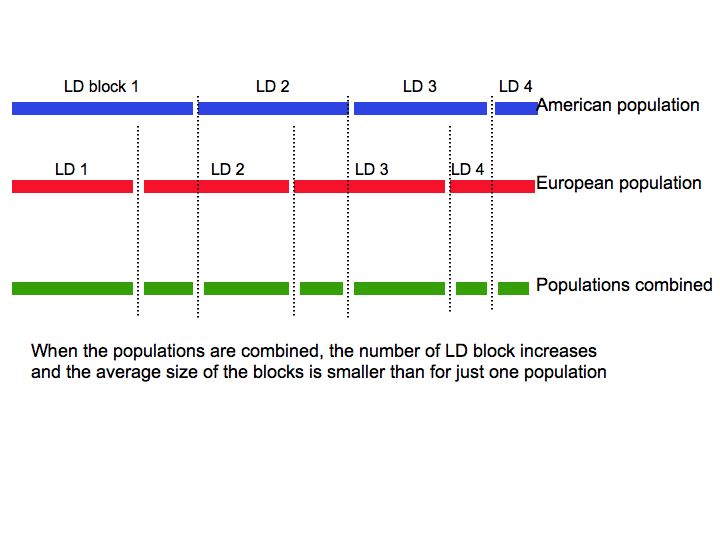

Supplement: Figure S1 — Using subtle population sub-structure to shape shorter-range patterns of haplotype and LD. Individuals in isolated dog populations (US or EU) with few or no exchange between them lead to a specific haplotype pattern and extent of LD. Population merged from both US and EU continent generated shorter-range patterns of haplotype, and thereby create shorter-range LD. (0.04 MB JPG) [file pone.0001324.s004.jpg]

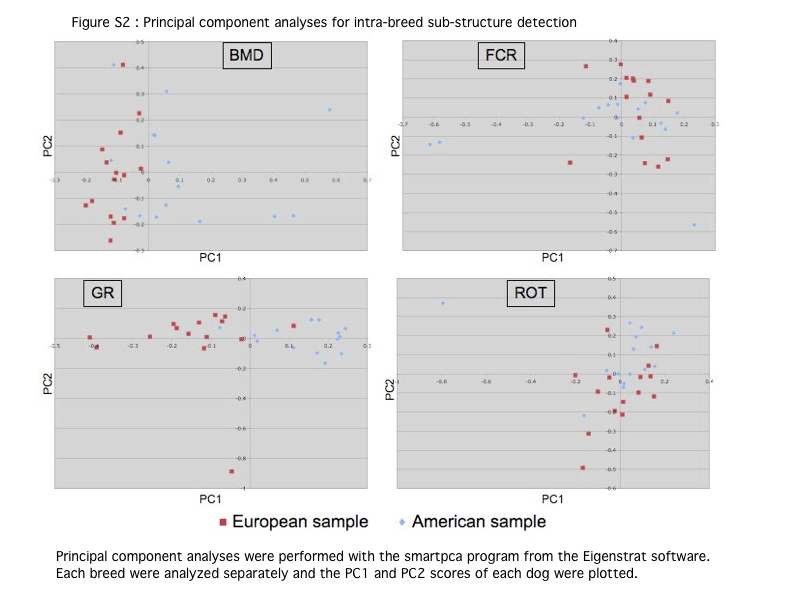

Supplement: Figure S2 — Principal component analyses were performed with the smartpca program from the Eigenstrat software. Each breed were analyzed separately and the PC1 and PC2 scores of each dog were plotted. Red squares represent European dogs and blue diamond US dogs. (0.18 MB TIF) [file pone.0001324.s005.tif]
